# Metabolic engineering of *Saccharomyces cerevisiae* to produce 1-hexadecanol from xylose

**DOI:** 10.1186/s12934-016-0423-9

**Published:** 2016-02-01

**Authors:** Weihua Guo, Jiayuan Sheng, Huimin Zhao, Xueyang Feng

**Affiliations:** Department of Biological Systems Engineering, Virginia Polytechnic Institute and State University, Blacksburg, VA 24061 USA; Department of Chemical and Biomolecular Engineering, University of Illinois at Urbana-Champaign, Urbana, IL 61801 USA

**Keywords:** Yeast, Promoter engineering, Evolutionary engineering, Fatty alcohols

## Abstract

**Background:**

An advantageous but challenging approach to overcome the limited supply of petroleum and relieve the greenhouse effect is to produce bulk chemicals from renewable materials. Fatty alcohols, with a billion-dollar global market, are important raw chemicals for detergents, emulsifiers, lubricants, and cosmetics production. Microbial production of fatty alcohols has been successfully achieved in several industrial microorganisms. However, most of the achievements were using glucose, an edible sugar, as the carbon source. To produce fatty alcohols in a renewable manner, non-edible sugars such as xylose will be a more appropriate feedstock.

**Results:**

In this study, we aim to engineer a *Saccharomyces cerevisiae* strain that can efficiently convert xylose to fatty alcohols. To this end, we first introduced the fungal xylose utilization pathway consisting of xylose reductase (XR), xylitol dehydrogenase (XDH), and xylulose kinase (XKS) into a fatty alcohol-producing *S. cerevisiae* strain (XF3) that was developed in our previous studies to achieve 1-hexadecanol production from xylose at 0.4 g/L. We next applied promoter engineering on the xylose utilization pathway to optimize the expression levels of XR, XDH, and XKS, and increased the 1-hexadecanol titer by 171 %. To further improve the xylose-based fatty alcohol production, two optimized *S. cerevisiae* strains from promoter engineering were evolved with the xylose as the sole carbon source. We found that the cell growth rate was improved at the expense of decreased fatty alcohol production, which indicated 1-hexadecanol was mainly produced as a non-growth associated product. Finally, through fed-batch fermentation, we successfully achieved 1-hexadecanol production at over 1.2 g/L using xylose as the sole carbon source, which represents the highest titer of xylose-based 1-hexadecanol reported in microbes to date.

**Conclusions:**

A fatty alcohol-producing *S. cerevisiae* strain was engineered in this study to produce 1-hexadecanol from xylose. Although the xylose pathway we developed in this study could be further improved, this proof-of-concept study, for the first time to our best knowledge, demonstrated that the xylose-based fatty alcohol could be produced in *S. cerevisiae* with potential applications in developing consolidated bioprocessing for producing other fatty acid-derived chemicals.

**Electronic supplementary material:**

The online version of this article (doi:10.1186/s12934-016-0423-9) contains supplementary material, which is available to authorized users.

## Background

Producing bulk chemicals from renewable resources could reduce strong dependence on petroleum and the damage to the environment [1, 2]. As important chemicals with a billion-dollar market globally [3, 4], fatty alcohols have been widely used to produce detergents, emulsifiers, lubricants, cosmetics, and have the potential to be used as fuels [5]. Currently, fatty alcohols are produced in two ways. One is direct extraction from natural plant oils [6], and the other is chemical synthesis from petrochemical sources. These methods have limitations due to either competition with the food supply, environmental concerns [7], or fast depletion of fossil sources [8].

Recently, with the development of metabolic engineering and synthetic biology, microbial production of fatty alcohols from renewable feedstock has been achieved successfully in both *Escherichia coli* [4, 9] and *Saccharomyces cerevisiae* [3, 10]. So far, the highest titer of fatty alcohols produced was 1.95 [11] and 1.1 g/L [10] by *E. coli* and *S. cerevisiae* respectively. A significantly higher titer of fatty alcohols was recently reported to be produced by *R. toloroides* [12]. In *E. coli*, fatty alcohols have been produced by introducing heterologous enzymes such as fatty acyl-CoA reductase (FAR) [13], carboxylic acid reductase (CAR) [14] together with aldehyde reductases (AR), or acyl-CoA reductase (ACR) together with AR [4, 15]. Compared to that in *E. coli*, the synthetic route (i.e., steps of enzymatic reactions) of fatty acyl-CoA is shorter in yeast, which allows more efficient conversion of carbohydrate substrates to fatty acids and fatty acid-derived biofuels [9]. Also, as a well characterized robust industrial host, yeast can grow under low pH and various harsh fermentation conditions [16]. Therefore, there has been an increasing interest on developing yeast, such as *S. cerevisiae*, as a cell factory for fatty acid-derived biofuel production. In *S. cerevisiae*, a mouse FAR has been expressed to produce 1-hexadecanol [10]. Through over-expression of acetyl-CoA carboxylase (ACC) and fatty-acyl-CoA synthases (FAS), the engineered *S. cerevisiae* strain produced 98.0 mg/L total fatty alcohol from 20 g/L glucose in batch culture in minimal medium [3]. Recently, by manipulating the structural genes in yeast lipid metabolism, tuning the regulation of phospholipid synthesis, and increasing the supply of key precursors, 1-hexadecanol was produced at 1.1 g/L using glucose as the carbon source in a fed-batch fermentation [10].

One of the limitations for current research on metabolic engineering to produce fatty acid-derived chemicals is that almost all of the achievements were based on glucose as the carbon source. To produce biofuels and biochemicals in a renewable manner, non-edible sugars such as xylose will be a more appropriate feedstock. Recently, engineering *S. cerevisiae* to utilize xylose is of great interest to the biofuel industry and could solve the major bottleneck in complete and efficient conversion of cellulosic sugars present in solubilized cell wall of plants into biofuels [17]. The sugar d-xylose, derived from hydrolysis of hemicellulose, is the second most abundant sugar in the plant cell wall consisting of up to 35 % of the total carbohydrate from lignocellulose biomass [18]. However, since the yeast *S. cerevisiae* cannot metabolize xylose, heterologous xylose utilization pathways need to be introduced into *S. cerevisiae* to achieve this objective. Two different pathways for the catabolism of d-xylose have been established in *S. cerevisiae*: the fungal xylose pathway consisting of xylose reductase (XR), xylitol dehydrogenase (XDH), and xylulose kinase (XKS) [17, 19–21], and the xylose pathway using the enzyme, xylose isomerase, to convert d-xylose directly into d-xylulose [22, 23], followed by the phosphorylation of d-xylulose to d-xylulose-5-phosphate. Recently, xylose isomerase has been successfully used in an industrial yeast strain [24].

In this study, we aim to engineer a *S. cerevisiae* strain that can efficiently convert xylose to fatty alcohols, by expressing a heterologous fungal xylose pathway into a 1-hexadecanol-producing *S. cerevisiae* strain that has been previously developed. We chose a *S. cerevisiae* strain, namely XF3, as our host since it has been engineered to produce 1-hexadecanol at over 1 g/L from glucose, and introduced XR, XDH, and XKS into XF3 to utilize xylose as the sole carbon source. Then, by applying combinatorial promoter engineering and evolutionary engineering, the production of 1-hexadecanol was enhanced by 171 %. Finally, over 1.2 g/L 1-hexadecanol was produced in a fed-batch fermentation using xylose as the sole carbon source, which is at the similar level when using the glucose as the carbon source [10]. To our best knowledge, it is the first time that yeast was engineered to use a pentose sugar for producing fatty acid-derived biofuels.

## Methods

### Yeast strains, media, and transformation

The yeast strains used in this study were derived from BY4742 (Table 1). Yeast and bacterial strains were stored in 25 % glycerol at −80 °C. *E. coli* DH5α strain was used to maintain and amplify plasmids, and recombinant strains were cultured at 37 **°**C in Luria–Bertani (LB) broth. Ampicillin at 100 μg/mL was added to the medium when required. Yeast BY4742 strains were cultivated in YPAD medium. Yeast cells were transformed with plasmids listed in Table 1 using the LiAc/PEG method as described previously [25]. To select the yeast transformants, a synthetic complete (SC) medium was used, which contains 0.17 % yeast nitrogen base, 0.5 % ammonium sulfate, and the appropriate amino acids dropout mix (MP Biomedicals, Solon, OH). A single colony was picked and cultivated in 5 mL SC medium containing 20 g/L glucose. The cells were cultivated at 30 °C in disposable culture tubes shaken at 250 rpm for 2 days.

**Table 1:** Plasmids and strains used in this study

Name	Description	Reference	
Plasmids used in this study			
pTaFAR_ACC1	pRS425-TEF1p-TaFAR-TEF1t-PGK1p-ACC1-HXT7t	[36]	
pYlACL	pRS423-TPI1p-YlACL1-TPI1t-TEF1p-YlACL2-TEF1t	[39]	
pXF3X01	pRS416-PDC1p(L)-csXR-ADH1t-TEF1p(L)-ctXDH-CYC1t-ENO2p(L)-ppXKS-ADH2t	This study	
pXF3X02	pRS416-PDC1p(L)-csXR-ADH1t-TEF1p(L)-ctXDH-CYC1t-ENO2p(M)-ppXKS-ADH2t	This study	
pXF3X03	pRS416-PDC1p(L)-csXR-ADH1t-TEF1p(L)-ctXDH-CYC1t-ENO2p(H)-ppXKS-ADH2t	This study	
pXF3X04	pRS416-PDC1p(L)-csXR-ADH1t-TEF1p(M)-ctXDH-CYC1t-ENO2p(L)-ppXKS-ADH2t	This study	
pXF3X05	pRS416-PDC1p(L)-csXR-ADH1t-TEF1p(M)-ctXDH-CYC1t-ENO2p(M)-ppXKS-ADH2t	This study	
pXF3X06	pRS416-PDC1p(L)-csXR-ADH1t-TEF1p(M)-ctXDH-CYC1t-ENO2p(H)-ppXKS-ADH2t	This study	
pXF3X07	pRS416-PDC1p(L)-csXR-ADH1t-TEF1p(H)-ctXDH-CYC1t-ENO2p(L)-ppXKS-ADH2t	This study	
pXF3X08	pRS416-PDC1p(L)-csXR-ADH1t-TEF1p(H)-ctXDH-CYC1t-ENO2p(M)-ppXKS-ADH2t	This study	
pXF3X09	pRS416-PDC1p(L)-csXR-ADH1t-TEF1p(H)-ctXDH-CYC1t-ENO2p(H)-ppXKS-ADH2t	This study	
pXF3X10	pRS416-PDC1p(M)-csXR-ADH1t-TEF1p(L)-ctXDH-CYC1t-ENO2p(L)-ppXKS-ADH2t	This study	
pXF3X11	pRS416-PDC1p(M)-csXR-ADH1t-TEF1p(L)-ctXDH-CYC1t-ENO2p(M)-ppXKS-ADH2t	This study	
pXF3X12	pRS416-PDC1p(M)-csXR-ADH1t-TEF1p(L)-ctXDH-CYC1t-ENO2p(H)-ppXKS-ADH2t	This study	
pXF3X13	pRS416-PDC1p(M)-csXR-ADH1t-TEF1p(M)-ctXDH-CYC1t-ENO2p(L)-ppXKS-ADH2t	This study	
pXF3X14	pRS416-PDC1p(M)-csXR-ADH1t-TEF1p(M)-ctXDH-CYC1t-ENO2p(M)-ppXKS-ADH2t	This study	
pXF3X15	pRS416-PDC1p(M)-csXR-ADH1t-TEF1p(M)-ctXDH-CYC1t-ENO2p(H)-ppXKS-ADH2t	This study	
pXF3X16	pRS416-PDC1p(M)-csXR-ADH1t-TEF1p(H)-ctXDH-CYC1t-ENO2p(L)-ppXKS-ADH2t	This study	
pXF3X17	pRS416-PDC1p(M)-csXR-ADH1t-TEF1p(H)-ctXDH-CYC1t-ENO2p(M)-ppXKS-ADH2t	This study	
pXF3X18	pRS416-PDC1p(M)-csXR-ADH1t-TEF1p(H)-ctXDH-CYC1t-ENO2p(H)-ppXKS-ADH2t	This study	
pXF3X19	pRS416-PDC1p(H)-csXR-ADH1t-TEF1p(L)-ctXDH-CYC1t-ENO2p(L)-ppXKS-ADH2t	This study	
pXF3X20	pRS416-PDC1p(H)-csXR-ADH1t-TEF1p(L)-ctXDH-CYC1t-ENO2p(M)-ppXKS-ADH2t	This study	
pXF3X21	pRS416-PDC1p(H)-csXR-ADH1t-TEF1p(L)-ctXDH-CYC1t-ENO2p(H)-ppXKS-ADH2t	This study	
pXF3X22	pRS416-PDC1p(H)-csXR-ADH1t-TEF1p(M)-ctXDH-CYC1t-ENO2p(L)-ppXKS-ADH2t	This study	
pXF3X23	pRS416-PDC1p(H)-csXR-ADH1t-TEF1p(M)-ctXDH-CYC1t-ENO2p(M)-ppXKS-ADH2t	This study	
pXF3X24	pRS416-PDC1p(H)-csXR-ADH1t-TEF1p(M)-ctXDH-CYC1t-ENO2p(H)-ppXKS-ADH2t	This study	
pXF3X25	pRS416-PDC1p(H)-csXR-ADH1t-TEF1p(H)-ctXDH-CYC1t-ENO2p(L)-ppXKS-ADH2t	This study	
pXF3X26	pRS416-PDC1p(H)-csXR-ADH1t-TEF1p(H)-ctXDH-CYC1t-ENO2p(M)-ppXKS-ADH2t	This study	
pXF3X27	pRS416-PDC1p(H)-csXR-ADH1t-TEF1p(H)-ctXDH-CYC1t-ENO2p(H)-ppXKS-ADH2t	This study	
pXF3XP	pRS416-PDC1p-csXR-ADH1t-TEF1p-ctXDH-CYC1t-ENO2p-ppXKS-ADH2t	This study	
pXF3XPi	pRS416-PDC1p*-csXR-ADH1t-TEF1p*-ctXDH-CYC1t-ENO2p*-ppXKS-ADH2t	[40]	

Name	Genotype	Plasmids	Reference
Strains used in this study			
BY4742	*MATα his3Δ1 leu2Δ0 lys2Δ0 ura3Δ0*		
XF3	BY4742 *ΔRPD3*: pTaFAR_ACC1, pYlACL		[36]
XF3XP	Same as XF3	pXF3XP	This study
XF3XPi	Same as XF3	pXF3XPi	This study
XF3X01	Same as XF3	pXF3X01	This study
XF3X02	Same as XF3	pXF3X02	This study
XF3X03	Same as XF3	pXF3X03	This study
XF3X04	Same as XF3	pXF3X04	This study
XF3X05	Same as XF3	pXF3X05	This study
XF3X06	Same as XF3	pXF3X06	This study
XF3X07	Same as XF3	pXF3X07	This study
XF3X08	Same as XF3	pXF3X08	This study
XF3X09	Same as XF3	pXF3X09	This study
XF3X10	Same as XF3	pXF3X10	This study
XF3X11	Same as XF3	pXF3X11	This study
XF3X12	Same as XF3	pXF3X12	This study
XF3X13	Same as XF3	pXF3X13	This study
XF3X14	Same as XF3	pXF3X14	This study
XF3X15	Same as XF3	pXF3X15	This study
XF3X16	Same as XF3	pXF3X16	This study
XF3X17	Same as XF3	pXF3X17	This study
XF3X18	Same as XF3	pXF3X18	This study
XF3X19	Same as XF3	pXF3X19	This study
XF3X20	Same as XF3	pXF3X20	This study
XF3X21	Same as XF3	pXF3X21	This study
XF3X22	Same as XF3	pXF3X22	This study
XF3X23	Same as XF3	pXF3X23	This study
XF3X24	Same as XF3	pXF3X24	This study
XF3X25	Same as XF3	pXF3X25	This study
XF3X26	Same as XF3	pXF3X26	This study
XF3X27	Same as XF3	pXF3X27	This study

* Mutated promoters used for ethanol production [40]

### Plasmid construction

**Fig. 1:**
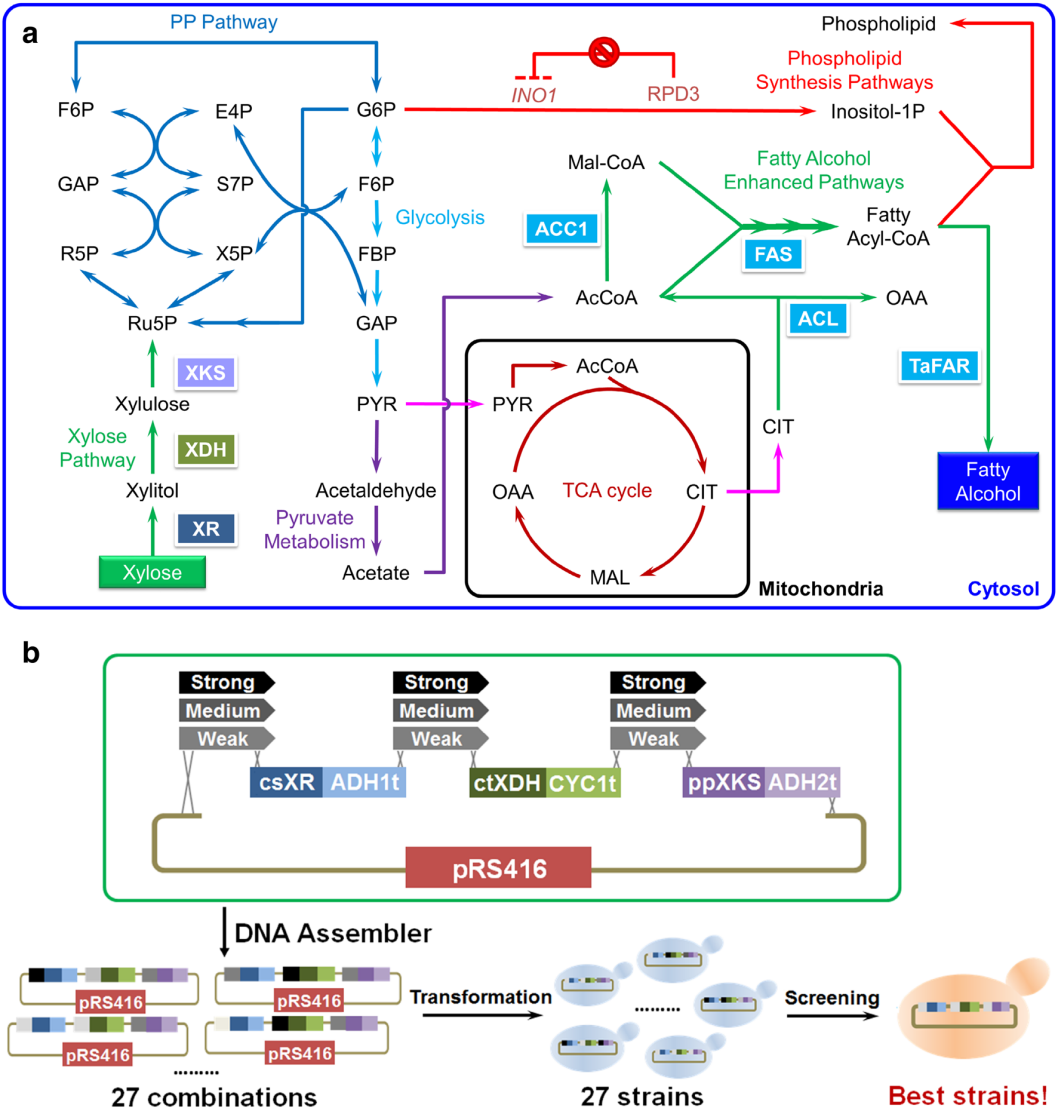
Overview of the approaches for xylose-based fatty alcohol production and improvement. **a** Scheme for the introduction of xylose utilization pathway to a fatty alcohol-producing *S. cerevisiae* strain. The xylose utilization pathway was constituted with three fungal enzymes, XR, XDH and XKS, from our previous study. This pathway has been confirmed to use xylose as the sole carbon source. The XF3 strain was selected from our previous study in which we over-expressed a bird FAR to produce the 1-hexdecanol and engineered the yeast lipid metabolism to further improve the fatty alcohol production. **b** Plasmid design for promoter engineering to further improve the xylose-based fatty alcohol production. We designed 27 different plasmids to exhaust all of the combinations of the promoters in front of XR, XDH, and XKS whose strengths were low, medium and high, respectively. The fatty alcohol production and growth behavior were monitored in these 27 recombinant strains

A yeast homologous recombination-based method, DNA assembler [26], was used to construct the recombinant plasmids. Briefly, DNA fragments sharing homologous regions to adjacent DNA fragments were co-transformed into *S. cerevisiae* along with the linearized backbone to assemble several elements in a single step [27]. Oligonucleotides used in this study were listed in Additional file 1: Table S1 and the recombinant plasmids constructed in this study were listed in Table 1. To construct the library for promoter engineering, the csXR was amplified with forward primer (XF_FP_csXR_ADH1t) and reverse primer (XF_RP_csXR_ADH1t); the ctXDH was amplified with forward primer (XF_FP_ TEF1p _CYC1t) and reverse primer (XF_RP_ctXDH_CYC1t); the ppXKS was amplified with forward primer (XF_FP_ppXKS_ADH2t) and reverse primer (XF_FP_ppXKS_ADH2t). The resulting PCR fragments have a 40 bp region homologous to constitutive yeast promoters and terminators, respectively. The constitutive yeast PDC1p promoters with different strengths for csXR were amplified with forward primer (XF_FP_PDC1p) and reverse primer (XF_RP_PDC1p) and using mutant PDC1p templates [28]. The different versions of TEF1p promoters for ctXDH and ENO2p promoters for ppXKS were achieved using the same methods. The DNA Assembler method was next used to construct the xylose utilization plasmids pRS416-PDC1p (L/M/H)-csXR-ADH1t-TEF1p(L/M/H)-ctXDH-CYC1t-ENO2p(L/M/H)-ppXKS-ADH2t with the proper combinations of each fragment (Fig. 1b). The sequences of all mutated promoters were listed in Additional file 1: Table S2.

### Determination of 1-hexadecanol production

The 1-hexadecanol was detected using a method previously described [3]. In general, for screening the 1-hexadecanol production in different strains, the engineered yeast strains were pre-cultured in 3 mL SC medium including all the appropriate nucleotides and amino acids, with 2 % glucose for 3 days until saturation. The cells were then centrifuged and washed twice with double-distilled water. The cell pellets were next inoculated into 5 mL fresh SC medium with 40 g/L xylose in disposable glass tubes overlaid with 10 % dodecane to prevent the evaporation of fatty alcohols and enrich the fatty alcohol in the organic layer to ease the measurement [3]. The concentrations of 1-hexadecanol were quantified at 48 h [3]. The glass tubes of yeast cultures were allowed to sit for 2 min until the organic layer could be clearly visualized. Then, 3 μL of dodecane was withdrawn from the organic layer and diluted by 100 times using ethyl acetate and analyzed by GC–MS (ShimadzuGC-MS-QP2010) with a DB-Wax column with 0.25 μm film thickness, 0.25 mm diameter, and 30 m length (Agilent Inc., Palo Alto, CA). Tridecane at a concentration of 2 mg/L was used as the internal standard. The GC program was as follows: an initial temperature of 50 °C was maintained for 1.5 min, followed by ramping to 180 °C at a rate of 25 °C/min. The temperature was then ramped to 250 °C at a rate of 10 °C/min, where the temperature was held for 3 min.

### Evolutionary engineering

To improve xylose utilization of the engineered strain, the optimized strain was cultured and serial-transferred into 50 mL of fresh SC medium with 40 g/L xylose as the sole carbon source in a closed 100 mL flask. The cells were grown until the early stationary phase (~3 days) and spread on SC-xylose plates. After 3-days growth, the biggest colony was inoculated into a fresh medium and grown until the early stationary phase. Then the cells were sub-cultured with 5 % inoculums in biological triplicates into fresh medium for the second round evolutionary engineering using the SC medium with 40 g/L xylose. The cells were grown for 3 days with typical OD_600_ in the range of 1.5–2.5. For each round of cell culture, the cell growth rate and fatty alcohol titer were measured using the method described above. We checked the plasmids intactness for each generation of the evolved strains by colony PCR, amplifying the cassettes for each gene, and confirming the intactness by DNA electrophoresis. All the plasmids were found to be intact (Additional file 1: Figure S1).

### Batch and fed-batch fermentation

Both XF3XP and XF3XP07 yeast strains were first grown in 100 mL SC medium including all the appropriate nucleotides and amino acids, with 20 g/L glucose for 2 days. Then, cells from 5 mL of culture were centrifuged, washed twice with double-distilled water, and inoculated into 5 mL fresh SC medium with 40 g/L xylose in glass disposable tubes overlaid with 0.5 mL dodecane for batch fermentation. The initial ODs were similar, i.e., 2.38 ± 0.05 and 2.45 ± 0.06, with no significant difference (*p* > 0.05). Samples were taken at various time points to measure the 1-hexadecanol concentration, OD_600_, and xylose concentration. At each time point, the glass tubes of yeast cultures were allowed to sit for 2 min until the organic layer could be clearly visualized. To measure the 1-hexadecanol concentration, 3 μL of dodecane was withdrawn from the organic layer and then diluted by 100 times using ethyl acetate followed by the analysis using the GC–MS protocol mentioned above. To monitor OD_600_, 20 μL of yeast culture was taken from the water layer and mixed with 180 μL of double-distilled water, followed by measuring the absorbance at 600 nm using a Biotek Synergy 2 Multi-Mode Microplate Reader (Winooski, VT). To measure the concentration of xylose, 100 μL of yeast culture was taken from the water layer and mixed with 900 μL of double-distilled water, which was then centrifuged at 13,000 rpm for 5 min. The supernatant was taken and analyzed by Shimadzu HPLC (Columbia, MD) equipped with an Aminex HPX-87H column (Bio-Rad, Hercules, CA) and Shimadzu RID-10A refractive index detector. The column was kept at 50 °C, and 5 mM sulfuric acid solution was used as a mobile phase with a constant flow rate of 0.6 mL/min. Each data point represents the mean of triplicate samples. In this discontinuous fed-batch fermentation, additional xylose (0.5 mL with concentration of 200 g/L) and dodecane (0.05 mL) were fed every 12 h. Samples were taken after the replenishment to measure 1-hexadecanol concentration, OD_600_, and xylose concentration using the similar methods as that for batch fermentation. The biological triplicates were implemented in both batch and fed-batch fermentation for all the strains.

## Results and discussion

### Constructing a xylose utilization pathway in a fatty alcohol-producing strain

In order to produce the xylose-based 1-hexadecanol, we first introduced the fungal xylose utilization pathway [29] into a 1-hexadecanol-producing *S. cerevisiae* strain, XF3 [10] (Fig. 1). The xylose utilization pathway was selected from our previous study [29], which included a XR from *Candida shehatae*, a XDH from *Candida tropicalis* and a XKS from *Pichia pastoris*. The XF3 strain produced 1-hexadecanol at over 1.1 g/L from glucose in *S. cerevisiae* as reported in our previous study [10]. The 1-hexadecanol production in XF3 was achieved by heterologously expressing a FAR from barn owls, over-expressing acetyl-CoA carboxylase (*ACC*1 gene), knocking out a negative regulator, *RPD3* gene, in phospholipid synthesis, and over-expressing ATP-citrate lyases (*ACL*1 gene and *ACL*2 gene) from *Yarrowia lipolytica* to enhancing the supply of cytosolic acetyl-CoA (Fig. 1a). By introducing the fungal xylose utilization pathway into XF3 strain, we successfully generated a *S. cerevisiae* strain (XF3XP) to produce the 1-hexadecanol from xylose as the sole carbon source at 0.4 g/L (Table 2). The xylose-based fatty alcohol titer was lower than the glucose-based 1-hexadecanol titer [10] and only 15 g/L xylose was consumed to produce 1-hexadecanol, indicating the xylose utilization could be a rate-limiting step for fatty alcohol production. We also introduced another fungal xylose pathway in which the promoter strengths of XR, XDH, and XKS were previously optimized to increase xylose-based ethanol production (XF3XPi, Table 2). We found that although 1-hexadecanol production could be increased to 0.48 g/L, the xylose utilization was even worse than the wild-type pathway with less than 5 g/L xylose consumed. This is possibly due to the fact that the regulatory mechanism adopted by *S. cerevisiae* to control xylose-based fatty alcohol production was different from that to control xylose-based ethanol production. Therefore, the metabolic engineering of *S. cerevisiae* for biofuel production is target-specific.

**Table 2:** Batch fermentation profiles of engineered *S. cerevisiae* strains

Strains	Xylose consumed (g/L)	Growth rate (h^−1^)	1-hexadecanol (g/L)	Ethanol (g/L)
XF3XP	14.9 ± 0.3	0.093 ± 0.009	0.40 ± 0.10	1.41 ± 0.32
XF3XPi	4.5 ± 0.4	0.096 ± 0.010	0.48 ± 0.09	0.48 ± 0.07
XF3XP07	7.8 ± 1.3	0.073 ± 0.007	0.79 ± 0.10	0.00 ± 0.00

### Promoter engineering to improve 1-hexadecanol production from xylose

**Fig. 2:**
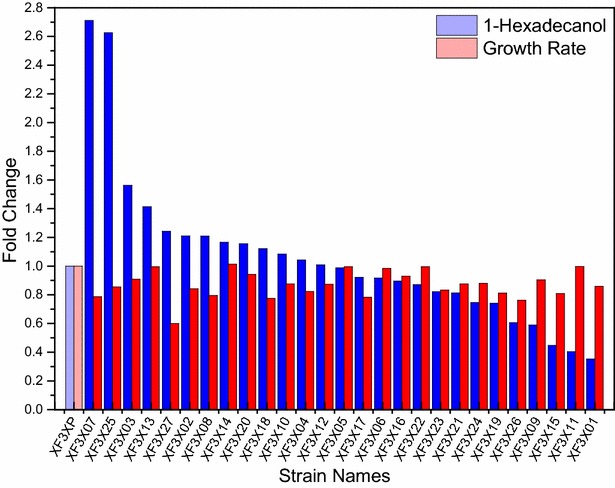
1-Hexadecanol produced and growth rates of engineered *S. cerevisiae* strains via promoter engineering. All the strains were cultured in the SC-xylose (4 %) medium for 48 h. The *bars with lighter color* were the values for the control strain (i.e., XF3XP) with the xylose utilization pathway using the native promoters

In order to further improve the 1-hexadecanol production, we implemented a synthetic biology approach called Customized Optimization of Metabolic Pathways by Combinatorial Transcriptional Engineering (COMPACTER) [28] to precisely control the gene expression levels of XR, XDH, and XKS. Basically, we chose three constitutive promoters, P_PDC1_, P_TEF1_, and P_ENO2_ to express XR, XDH and XKS genes, respectively. For each of the constitutive promoters, we mutated the original promoters to create a promoter library with varying strengths. We then selected promoters with high, medium, and low strengths (Additional file 1: Figure S2) for P_PDC1_, P_TEF1_, and P_ENO2_, respectively, and constructed totally 27 synthetic xylose pathways (3 × 3 × 3 = 27) in *S. cerevisiae* with all of the promoter combinations of P_PDC1_, P_TEF1_, and P_ENO2_ with different strengths (Fig. 1b; Table 1). We next compared the growth rates and 1-hexadecanol titers of all the recombinant *S. cerevisiae* strains to that of the control strains, XF3XP (Fig. 2). It is worth noticing that the purpose of combinatorial promoter screening was to find the strain with the highest fatty alcohol production from xylose instead of the best xylose utilization strain. Therefore, we did not measure the xylose utilization rates here. We found that the growth rates of most of the promoter-engineered strains were reduced to some extent, and the 1-hexadecanol production for most of the recombinant strains was not significantly improved. However, strain XF3X07 and XF3X25 produced 1-hexadecanol at 171 and 140 % higher than that of the control strains with a slightly deceased growth rates (0.073 h^−1^ and 0.080 h^−1^) compared with the growth rate of the control strain (0.093 h^−1^). Both XF3X07 and XF3X25 used a high-level TEF1 promoter to express XDH and a low-level ENO2 promoter to express XKS. Nevertheless, XF3X07 used a low-level PDC1 promoter to express XR while XF3X25 used a high-level PDC1 promoter. This discovery is consistent with previous studies showing that the XDH enzymes were rate-limiting steps in converting xylose to biomass and ethanol [30, 31]. Interestingly, despite of higher titer of 1-hexadecanol in XF3X07 compared to XF3XPi, the xylose-based 1-hexadecanol yields were similar in XF3XP07 and XF3XPi (*p* > 0.1). This indicated that the combinatorial promoter engineering mainly improved the xylose uptake rate instead of optimizing the host pathways to improve the conversion of xylose to 1-hexadecanol.

We correlated the strengths of promoters for XR, XDH, and XKS with the two measured parameters, 1-hexadecanol concentrations and growth rates (Additional file 1: Figure S3). No correlation was observed between promoter strengths and 1-hexadecanol concentrations. Neither did we find the correlation between promoter strengths and growth rates. We also correlated 1-hexadecanol concentrations and growth rates, but found no correlation between them either (Additional file 1: Figure S4). Therefore, it is unfeasible to solely use the results of promoter screening to make predictions on the choice of promoters that should be used for xylose-based production of 1-hexadecanol. This is because the introduction of xylose pathways would trigger the global metabolic rewiring, as we found previously when investigating metabolic responses to different xylose utilization pathways via ^13^C metabolic flux analysis [32]. This global metabolic rewiring involves the reprogramming of not only xylose pathway itself but also the downstream pathways, which made the xylose metabolism too complex to be correlated with the activity of xylose utilization pathway itself.

### Evolutionary engineering to improve 1-hexadecanol production from xylose

**Fig. 3:**
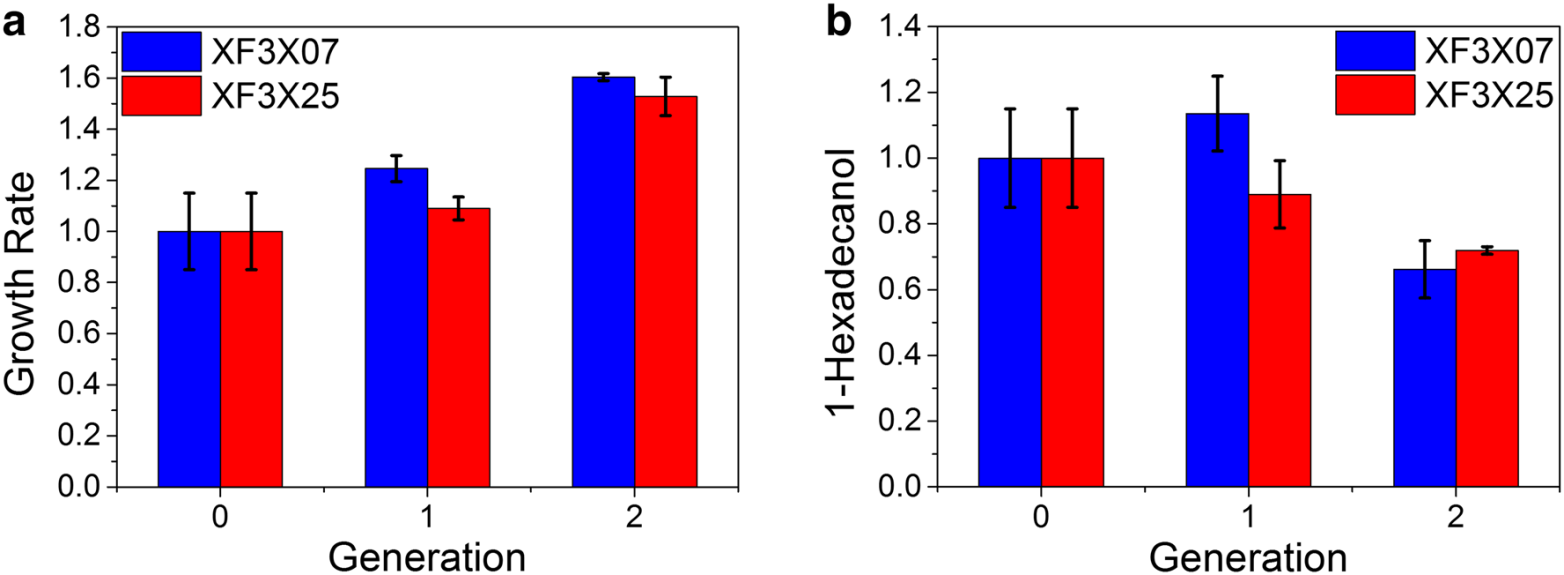
Evolutionary engineering of XF3X07 and XF3X25. 1-hexadecanol production (**a**) and growth rates (**b**) of the XF3X07 and XF3X25 in each round were normalized with 1-hexadecanol titer and growth rates of the XF3X07 and XF3X25 in round zero, respectively

We next chose XF3X07 and XF3X25 as our target strains for further evolutionary engineering to improve the 1-hexadecanol production. Evolutionary engineering has been widely used to improve the pentose utilization and xylose-based ethanol production in *S. cerevisiae* successfully [33–35]. Considering the poor xylose uptake in our engineered strains, we implemented the evolutionary engineering to investigate whether fatty alcohol production is growth-associated, and if so, to further improve the xylose-based fatty alcohol production. Similar as the study of combinatorial promoter screening, our goal of evolutionary engineering is to seek a yeast strain that could produce fatty alcohols from xylose as much as possible. Therefore, we did not measure the xylose utilization rates. In general, we serially transfer the strain XF3X07 and XF3X25 to synthetic medium with 40 g/L xylose twice. Namely, the optimized strain was the second generation evolved from the wild-type strain. We found that the growth rates of two strains increased gradually (~25 and ~ 35 %) for every round as expected. However, such increase was associated with reduced 1-hexadecanol production. For example, the highest growth rate was reached for both XF3X07 and XF3X25 with the lowest titer of 1-hexadecanol in the second round (Fig. 3). The growth rates of the evolved strains in the last round were significantly increased for XF3XP07 and XF3XP25 (*p* < 0.05). However, the 1-hexadecanol productions were not significantly changed (*p* > 0.05). Such discrepancy indicated that 1-hexadecanol, unlike ethanol, was not a growth-associated product. Since evolutionary engineering selects the mutant strain with higher growth rate, the 1-hexadecanol production failed to be further improved via adaptive evolution due to the de-coupling between the cell growth rate and the fatty alcohol production. In addition, we applied flux balance analysis to calculate the ATP, NADH, and NADPH synthesis under different 1-hexadecanol productions (Additional file 1: Figure S5). We found that the NADPH and ATP synthesis were positively correlated with 1-hexadecanol production, while NADH synthesis did not change too much with the 1-hexadecanol synthesis. Overall, the evolutionary engineering approach would be helpful to improve the cell growth and growth-associated products such as ethanol, but not for non-growth associated products such as fatty acid-derived chemicals.

### Batch and fed-batch fermentation for 1-hexadecanol production

With XF3XP07 as our best strain to produce xylose-based 1-hexadecanol, we next characterized its 1-hexadecanol production using batch and fed-batch fermentation. In batch fermentation, we found that 0.79 g/L 1-hexadecanol was produced from 7.8 g/L xylose, with a cell growth rate at 0.073 h^−1^ (Table 2). This 1-hexadecanol titer of XF3XP07 is significantly higher than the ones of XF3XP and XF3XPi strain (*p* < 0.05). More interestingly, comparing the xylose uptakes of the XF3XP and XF3XPi, we found the XF3XP strain consumed three-fold more xylose than the XF3XPi strain. This additional xylose was mainly used to produce more ethanol in XF3XP strains (Table 2). In addition, we have measured the accumulation of intracellular 1-hexadecanol, which was less than 5 % of the extracellular concentration of 1-hexadecanol from the organic layer. Such low accumulation is consistent with several previous studies when yeast was cultured with an organic layer [36], although it is also reported that *S. cerevisiae* strains could accumulate a large amount of fatty alcohols intracellularly when cultured without the organic layer [37].

**Fig. 4:**
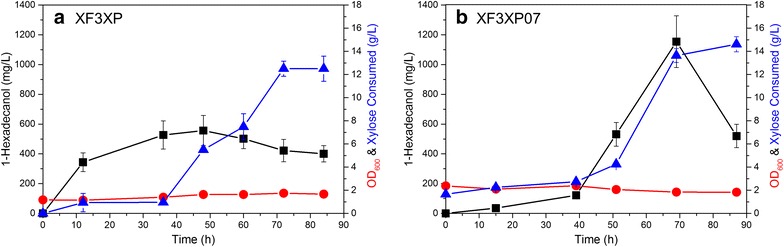
Fed-batch fermentation of xylose-based 1-hexadecanol production by **a** XF3XP and **b** XF3XP07. Ethanol was detected as the only byproduct other than 1-hexadecanol. *Black square* the 1-hexadecanol concentration; *blue triangle* the xylose consumed; *red dot* OD_600_

In fed-batch fermentation, we used resting cells for fermentation, i.e., the cell density was kept at high level to prevent using the xylose to produce biomass. Although the fermentation at high cell density might limit the oxygen supply for the fermentation, which is an important factor for the optimal expression of the xylose pathway genes [38], the marginal net growth rate of yeast cells could be more important in fed-batch fermentation because it was found in this study that fatty alcohol production was not growth-associated and hence by removing the biomass production, yeast cells could serve as biocatalysts to convert xylose to 1-hexadecanol with high efficiency. We found that a long lag phase lasting around 40 h in the fed-batch fermentation, which could be due to the repression of residue glucose from the inoculum since we cultured XF3XP and XF3XP07 with 20 g/L glucose before transferring the cells into the medium with xylose, and hence cells needed a long time to accustom to xylose from glucose (Fig. 4). For the XF3XP strain, 1-hexadecanol has been produced quickly with a low xylose consumption, and achieved ~0.6 g/L of 1-hexadecanol at 48 h (Fig. 4a). For the XP3XP07 strain, after the long lag phase, 1-hexadecanol was produced rapidly with the increased xylose uptake and reached the highest titer of 1-hexadecanol at 1.2 g/L at the 69 h (Fig. 4b). However, when continuing the fed-batch fermentation for both strains, both the 1-hexadecanol concentrations and xylose uptake rates were decreased. The observed low xylose consumption rate accompanied with the decrease of OD_600_ suggested a starvation because of the incapability to further uptake the carbon substrate and the probable limitation by other nutrients such as nitrogen and phosphate after the 50 h of the fermentation. In our previous study [10], we found that fatty alcohols could be taken up by *S. cerevisiae,* which could be the reason for the decreased fatty alcohol production when xylose utilization became limited.

Comparing the xylose-based fatty alcohol production to the glucose-based one in the previous study, the similar titer of fatty alcohol from fed-batch fermentation has been observed, demonstrating the successful integration of xylose utilization pathway and the fatty alcohol production pathway. However, the yields of xylose-based fatty alcohols in both batch (0.10 ± 0.02 g/g) and fed-batch fermentation (0.08 ± 0.01 g/g) were much higher than that of glucose-based ones (~0.03 and <0.01 g/g), respectively. The theoretical maximum yields through this production pathway from xylose and glucose were ~0.34 and ~0.35 (g/g), respectively. In this case, the yield from xylose reached nearly one-third of the theoretical yield, while the yield from glucose only reached less than 10 % of the theoretical yield. The bypass of ethanol production when feeding xylose instead of glucose likely attributed to the high yield of xylose-based 1-hexadecanol, which could divert more carbons to be utilized in the fatty alcohol production rather than ethanol production.

## Conclusion

A fatty alcohol-producing *S. cerevisiae* strain was engineered in this study to produce 1-hexadecanol from xylose. To achieve this, a xylose utilization pathway consisting of XR, XDH and XK was heterologously expressed in *S. cerevisiae*, followed by optimization of the xylose-based fatty alcohol production through promoter engineering and evolutionary engineering to improve 1-hexadecanol production by 171 %. Through fed-batch fermentation, the highest titer of 1-hexadecanol reached 1.2 g/L with xylose used as the sole carbon source. Although the xylose pathway we developed in this study was still not optimal, this proof-of-concept study, for the first time to our best knowledge, indicated that the xylose-based fatty alcohol could be achieved in *S. cerevisiae* with potential applications in developing consolidated bioprocessing for producing fatty acid-derived chemicals.

## Additional file

10.1186/s12934-016-0423-9 Primers used in this study. **Table S2.** Promoters used in this study. **Figure S1.** DNA electrophoresis confirmed that all of the cassettes in both xylose pathway and 1-hexadecanol pathway existed in the evolved strains. **Figure S2.** The strengths of PDC1, TEF1, and ENO2 promoters in front of EGFP. **Figure S3.** Correlations between the promoter strengths in front of XR, XDH and XKS in xylose utilization pathways and the growth rates (blue dots) as well as 1-hexadecanol concentrations (red dots). **Figure S4.** Correlation between the growth rates and the 1-hexadecanol titers for the combinatorial promoter engineering. **Figure S5.** Flux balance analysis revealed correlation between the 1-hexadecanol production and ATP (A), NADH (B), NADPH (C), and growth rate (D)

## Abbreviations

ACC: acetyl-CoA carboxylase
ACL: ATP-citrate lyase
AcCoA: acetyl-CoA
ACR: acyl-CoA reductase
AR: aldehyde reductases
CAR: carboxylic acid reductase
CIT: citrate
E4P: erythrose 4-phosphate
F6P: fructose 6-phosphate
FAS: fatty-acyl-CoA synthase
FBP: fructose 1,6-bisphosphate
G6P: glucose 6-phosphate
GAP: glyceraldehyde 3-phosphate
INO1: inositol-3-phosphate synthase
MAL: malate
OAA: oxaloacetate
PYR: pyruvate
R5P: ribose 5-phosphate
Ru5P: ribulose 5-phosphate
S7P: sedoheptulose 7-phosphate
TCA cycle: tricarboxylic acid cycle
X5P: xylulose 5-phosphate
XR: xylose reductase
XDH: xylitol dehydrogenase
XKS: xylulose kinase
