# Supplementary material for: Metabolic engineering of Saccharomyces cerevisiae to produce 1-hexadecanol from xylose
Source: Microb Cell Fact. 2016 Feb 1;15:24. doi: 10.1186/s12934-016-0423-9 (PMC4736120; doi:10.1186/s12934-016-0423-9)
Supplement: Supplementary file 1 — 10.1186/s12934-016-0423-9 Primers used in this study. Table S2. Promoters used in this study. Figure S1. DNA electrophoresis confirmed that all of the cassettes in both xylose pathway and 1-hexadecanol pathway existed in the evolved strains. Figure S2. The strengths of PDC1, TEF1, and ENO2 promoters in front of EGFP. Figure S3. Correlations between the promoter strengths in front of XR, XDH and XKS in xylose utilization pathways and the growth rates (blue dots) as well as 1-hexadecanol concentrations (red dots). Figure S4. Correlation between the growth rates and the 1-hexadecanol titers for the combinatorial promoter engineering. Figure S5. Flux balance analysis revealed correlation between the 1-hexadecanol production and ATP (A), NADH (B), NADPH (C), and growth rate (D) [file 12934_2016_423_MOESM1_ESM.docx]

**Supplementary materials**

**Table S1**. Primers used in this study

| **Name** | **Primer sequence** |
| --- | --- |
| XF_FP_csXR_ADH1t | ATGAGCCCAAGCCCAATTCC |
| XF_FP_ctXDH_CYC1t | ATGACTGCAAACCCATCATT |
| XF_FP_ppXKS_ADH2t | ATGGTTACCAAAGAAATCCA |
| XF_RP_csXR_ADH1t | GCATGCCGGTAGAGGTGTGG |
| XF_RP_ctXDH_CYC1t | GCAAATTAAAGCCTTCGAGC |
| XF_RP_ppXKS_ADH2t | CTCACTAAAGGGAACAAAAGCTGGAGCTCCACCGCGGTGGAGCTACTAATAGGATAAATT |
| XF_FP_PDC1p | AACGACGGCCAGTGAGCGCGCGTAATACGACTCACTATAGCATGCGACTGGGTGAGCATA |
| XF_FP_TEF1p | CATGAGGTCGCTCTTATTGACCACACCTCTACCGGCATGCATAGCTTCAAAATGTTTCTA |
| XF_FP_ENO2p | GCTTGAGAAGGTTTTGGGACGCTCGAAGGCTTTAATTTGCGTGTCGACGCTGCGGGTATA |
| XF_RP_PDC1p | CGTTGTTCAACTTGAAAGCTGGAATTGGGCTTGGGCTCATTTTGATTGATTTGACTGTGT |
| XF_RP_TEF1p | CGTCAACTTTGTTAAGAACTAATGATGGGTTTGCAGTCATTTTGTAATTAAAACTTAGAT |
| XF_RP_ENO2p | TCATTGCTGAATTTCTATCTTGGATTTCTTTGGTAACCATTATTATTGTATGTTATAGTA |

**Table S2.** Promoters used in this study

| **Name** | **Promoter Strength** | **Promoter sequence** |
| --- | --- | --- |
| TEF1p(H) | Strong | ATAGCTTCAAAATGTTTCTACTCCTTTTTTACTCTTCCGGATTTTCTCGGACTCCGCGCATCGCCGTACCACCTCAAAACACCCAAGCACAGCATACTCAATTCCCCCTCTTTCTTCCTCTAGGGTGTCGTTAATTACCCGTACTAAAGGTTTGGAAAAGAAGAAAGAGACCGCCTCGTCTCTCTTTCTTCGTCGAAAAAGGCAATAAAAATTTTTATTACGTTTCTTTTTCTTGAAAATTTTCTTTTTTGATTTTTCTCTCTTTCGATGACCTCCCATTGATATTTAAGTCATTAAATGGTCTTCAATTTCTCAAGTTTCAGTTTCATTTTTCTTATTCTATTACGACTCTTTTTACCTCTTGCTCATTAGAAAGAAAGCATAGCAATCTAATCTAAGTTTTAATTACAAA |
| TEF1p(M) | Medium | ATAGCTTCAAAATGTTTCTACTCCTTTTTTACTCTTCCAGATTTTCTCGGACTCCGCGCACCGCCGTACCACTTCAAAACACCCAAGCACAGCATACTAAATTCCCCCTCTTTCTTCCTCTAGGGTGTCGTTAATTACCCGTACTAAAGGTTTGGAAAAGAAAAAAGAGACCGCCTCGTTTCTTTTTCTTCGTCGAAGAAGGCAATAAAAATTTTTATCACGTTTCGTTTTCTTGAAAACTTTTCTTTTTGATTCTTTTCTCTTTCGATGACCTCCCATTGATATTTAAGTTATTAGATGGTCTTCAATTTCTCAAGTTTCAGCTCCATTTTTCTTGTTCTATTACAACTTTTTTTACTTCTTGCTCATTAGAAAGAAAGCATAGCAATCTAATCTAAGTTTTAATTACAAA |
| TEF1p(L) | Low | ATAGCTTCAAAATGTTTCTACTCCTTTTTTACTCCTCCAGACTTCCTCGGACTCCGCGCATCGCCGTACCACTTCAAAACACCCAAGCACAGCATACTAAATTCCCCCTCTTTCTTCCTCTAGGGTGTCGTTAATTACCCGTACTAAAGGTTTGGAAAAGAAAAAAGAGACCGCCTCGTTTCTTTTCCTTCGTCGAAAAAGGCAATAGAAATTTTTATCACGTTTTTCCTTCTTGAAAATCTTTTTTTTTGATTTTTTTCTCTTTCGATGACCTCCCATTGACATTTAAGTTATTAAATGGTCTTCAATTTCTCAAGTTTCAGTTTCATTTTTCTTGTTCTATTACAACTTTTTTTACTTCTTGCTCATTAGAAAGAAAGCATAGCAATCTAATCTAAGTTTTAATTACAAA |
| TEF1p* | (Mutated) | ATAGCTTCAAAATGTTTCTACTCCTTTTTTACTCTTCCAGATTTTCTCGGACTCCGCGCACCGCCGTACCACTTCAAAACACCCAAGCACAGCATACTAAATTCCCCCTCTTTCTTCCTCTAGGGTGTCGTTAATTACCCGTACTAAAGGTTTGGAAAAGAAAAAAGAGACCGCCTCGTTTCTTTTTCTTCGTCGAAGAAGGCAATAAAAATTTTTATCACGTTTCGTTTTCTTGAAAACTTTTCTTTTTGATTCTTTTCTCTTTCGATGACCTCCCATTGATATTTAAGTTATTAGATGGTCTTCAATTTCTCAAGTTTCAGCTCCATTTTTCTTGTTCTATTACAACTTTTTTTACTTCTTGCTCATTAGAAAGAAAGCATAGCAATCTAATCTAAGTTTTAATTACAAA |
| ENO2p(H) | Strong | GTGTCGACGCTGCGAGTATAGAAAGGGCTCTTTACTCTATAGTACCTCCTCGCTCGGCATCTGCTTCCTCCCAAAGATGAGCGCGGCGTCATGTCACTAACGACGTGCACCAACTTGCGGAAAGTGGAATCCCGTTCCAAAACTGGCATCCACTAATTGATACATCTACACACCGCACGCCTTTTTTCTGAAGCCCACTTTCGTGGACTTTGCCATATGCAAAATTCATGAAGTGTGACACTGAGTCAGCATACACCTCACTAGGGTAGTTTCTTTGGCTGTATTGATCATTTGATTCATCGTGGTTCATTAATTTTTTTTCTCCACTGCTTTCTGGCTTTGATCTTACTATCATTTGGATTTTTGTCGAAGGTTGTAGAATTGTGTGTGACAAGTGGCACCAAGCATATATAAAAAAAAAAAGCATTATCTTCCCACCAGAGTTGATTGTTAAAAACGTATTTATAGCAAACACAATTGTGATTAATTCTTATTTTGTATCTTTTCTTCCCTTGTCTCAATCTTTTATTTTCATTTTATTTTCCTTCTCTTAGTTTCTTTCATAACACCAAGCAACTAATACTATAACGTACAATAATA |
| ENO2p(M) | Medium | GTGTCGACGCTGCGGGTATAGAAAGGGTTCTTTACTCTATAGTACCTGCTCGCTCAGCACCTGCTTCTTCCCAAAGATGAACGCGGCGTTATGTCACTAACGACGTGCACCAACTTGCGGAAAGTGGAATCCCGTTCCAAAACTGGCATCCACTAATTGATACACCTACACACTGCACGCCTTTTTTCCGAAGCCCACTTCCGTGGACTTTGCCATATGCAAAATTCATGAAATGTGATACCAAGTCAGCATACACCTCACTAGGGCAGTTTCTTTGGTTGTATTGATCATTTGGTTCATCGCGGTTCATTAATTTTTTTTCTCCATTGCTTTCTGGCTTTGATCTTACTATCATTTGGATTTTTGTCGAAAGTTGTAGAATTGTATGTGACAAGTGGCACCAAGCATATATAAAAGAAAAAAAGCATCATCTTCCTACCAGAGTTGATTGTTAAGAACGTATTTATAGCAAACGCAGTTGTAGTTAATTCTTATTTTGTATCTTTTCTTCCCTTGTCCCGATCTTTTATTTTTATTTTATCTTTCTTTTCTTAGTTTCTGTCATAGCACCAAGCAACTAATACTATAACGTACAATAATA |
| ENO2p(L) | Low | GTGTCGACGCTGCGGGTATAGAAAGGGTTCTTGACTCTATAGTACCTCCTCGCTCAGCATCTGCTTCTTCCCACAGATGAACGCGGCGTTATGTCACTAACGACGTGCACCAACTTGCGGAAAGTGGAATCCCGTTCCAAAACTGGCATCCACGAATTGATAGATCTACACACCGCACGCCTTTTTTCTGAAGCCCACCTTCGTGGACTTTGCCATACGCAGAATTCGTGAAGTGTGATACCAAGTCAGCATACACCTCACCAGGGTAGTCTCTTTGGTTGTATTGATCATTTGGTTCATCGTGGTTCATTAATTTTTTTTCTCTATTGCTTTCTGGCTTTGATCTTACTATCATTTGGATTCTTGTCGAAGGTTGTAGAATTGTATGTGACAAGTGGCACCAAGCATATATAAAAAAAAAAAGCATTATCTTCCTACCAGAGTTGATTGTTAAAAACGTATTTATAGCAAACGCAATTGTAATTAATTCTTATTTTGTGTCTTTTCTTCCCTTGTCTCAATCTTTTATCTTTGTTTTATTCTTCTTTTCTTAGTTTCCTTCATAACACCAAGCAACTAACACTATAACATACAATAATA |
| ENO2p* | (Mutated) | GTGTCGACGCTGCGGGTATAGAAAGGGTTCTTTACTCTATAGTACCTCCTCGCTCAGCATCTGCTTCTTCCCAAAGATGAACACGGCGTTATGCCACTAACGGCGTGCACCGACTTGCGGAAAGTGGAATCCCGTTCCAAAACTGGCATCCACTAATTGATACATCTACACACCGCACGCCTTTTCTCTGAAACCCACTTTCGTGGACTTTGCCATATGCAAAATTTATGAAGTGTGATACCAAGTCAGCATACACCTCACTAGGGTAGTTTCTTTGGTTGTGTTGATCATTTGGTTCATCGTGGTTCATTAATTTTTTTTCTCCGTTGCTTTCTGGCCTTGATCTTACTATCATTTGGATTTTTGTCGAAGGTTGTAGAATTGTATGTGACAAGTGGCACCAAGCATATATAAAGAAAAAAAGCATTATCTTCCTACCAGAGTTGACTGCTAAAAACGTATTTATAGCAAACGCAATTGTAATTGATTCTTATTTTGTATCTTTTCTTCCCTTGTCTCAATCTTTTATTTTTATTTTATTTTTCTTTTCTTAGTTTCTTTCATAACACCAAGCAACTAATACTATAACATACAATAATA |
| PDC1p(H) | Strong | CATGCGACTGGGTGAGCATATGTTCCGCTGGTGTGATGTGCAAGATAAACAAGCAAGGCAGAAACTAACTTCTTCTTCATGTAATAAACACACCCCGCGTTTATTTACCTATCTCTAAACTTCAACACCTTATATCATAACTAGTGTTTCTTGAGGTAAGCACACTGCACCCATACCTTCCTTAAAAACGTAGCTTCCAGTTTTTGGTGGTTCCGGCTTCCTTCCCGATTCCGCCCGCTAGACGCATGTTTTTGTTGCCTGGCGGCATTTGTAAAGTGCATAACCTATGCATTTAAAAGATTATGCATGCTCTTCTGACTCTTCGTGTGATGAGGCTCGTGGAAAAAATGAATAATTTATGAATTTGAGAACAATTTTGTGTTGTTACGGTATTTTACTATGGAATAATCAATCAATTGAGGATTTTATGCAAATATTGTTTGAATATTTTTCCGACCCTTTGAGTACTTTTCTTCATAATTGCATAATATTGTCCGCTGCCCCTTTTTCTGTTAGACGGTGTCTTGATCTACTTGCTATCGTTCAACACCACCTTATTTTCTAACTATTTTTTTTTTAGCTCATTTGAATCAGCTTATGGTGATGGCACATTTTTGCATAAACCTAGCTGTCCTCGTTGAACATAGGAAAAAAAAAATATATAAACACGGCTCTTTCACTCTCCTTGCAATCAGATTTGGGTTTGTTCCCTTTATTTTCATATTTCTTGTCATATTCCTTTCTCAATTATTATTTTCTACTCATAACCTCACGCAAAATAACACAGTCAAATCAATCAAA |
| PDC1p(M) | Medium | CATGCGACTGGGTGAGCATATGTTCCGCTGATGTGATGTGCAAGATAAACAAGCAAGGCAGAAGCTAACTTCTTCTTCATGTGATAAACACACCCCGCGCTTATTTACCTATCTCTAAACCTCAACACCTTGTATCGTAACTAATATTTCTTGAGATAAGCACACTGCACCCATACCTTCCTTAAAAACGTAGCTTCCAGTTTTTGGTGGTTCCGGCTTCCTTCCCGATTCCGCCCGCTAAACGCATGTTTTTGTTGCCTGGTGGCATTTGCAAAATGCATAACCTATGCATTTAAAAGATTATGTATGCTCTTCTGACTTTTCGTGTGATGAGGCTCGTGGAAAAAATGAGTAATTTATGAACTTGAGAACAATTTTGTGTTGTTACGGTACTTTACTATGGAATAATCAATCAATTGAGGATTTTGTGTAAATATCGTTTGAATATTTCTCCGACCCTTTGAGTACTTTTCTTCATAATTGCATAATATTGTCCGCTGCCCCTTTTTCTGTTAGACGGCGTCTCGATCTACTTGCTGTCGTTCAACACCACCTTATTTTCTAACTATTTTTTTTTTAGCTCATTTGAATCGGCTTATGGTGATGGCACGTTTTTGCATAAACCTAGCTGTCCTCGTTGAACATAGGAAAAAAAAATATATAAACAAGGCTCTTTCACTCTCCTTGCAATCAGATTTGGGTTTGTTCCCTTTATTTTCATATTTCTTGCCATATTCCTTTCTCAATTATTATTTTCTACTTATAACCTCACGCAAAATAGCACAGTCAAATCAGTCAAG |
| PDC1p(L) | Low | CATGCGACTGGGTGAGCATATGTTCCGCTGATGTGATGTACAAGATAAACAAGCAAGGCAGAAACTAACTTCCTCTTCATGTAATAAACACACCCCGCGTTTATTTACCTATCTCTAAACTTCAACCCCCTATATCATAACTAATATTTCTTGAGATAAGCACACTGCACCCATACCTTTCTTAAAAGCGCAGCTTCCAGTTTTTGGTGGTTCCGGCTTCCTTCCCGATTCCGCCCGCTAAACGCATATTTTTGCTGCCTGGTGGCATTTGCAAAATGCATAACCTACGCATTTAGAAGATTATGTATGCTCTTTTGATTTTTCGCGTGATGAGGCTCGTGGAGAAAATGGATAATTTATGAATTTGAGAACAATTTCATGTTGTTACGGTATTTTACTATGGAATAATCAATCAATTGAGGATTTTATGCAAATATCGTTTGAATATCTTTCCGACCCTTCGAGTACTTTTCTTCATAATTGCATAATATTGTCCGCAGCCCCTTTTTCTGTTAGACGGTACCTTGATCTACTTGCTATCGTTCAACACCACCTTATTTTCTAACTATTTCTTCTTAGCTCATTTGAATCAGCCTGTGGTGATGGCACATTTTTGCACAAACCTAGCTGTCCTCGTTGAACATAGGAGGAAAAAACATATAAGCAGGGCTCTTTCACTCTCCTTGCAATCAGATTTGGGTTTGTTCCCTTTATTTCCATGCCTCTTGTCATATTCCTTTCTCAATTATTATTTTCTACTCATAACCTCACGCAAAATAACACAGTCAAATTAACCAAA |
| PDC1p* | Mutated | CATGCGACTGGGTGAGCATATGTTCCGCTGATGTGGTGTGCAAGATAAACAAGCAAGGCAGAAGCTAACTTCTTCTTCATGTGATAAACACACCCCGCGCTTATTTACCTATCTCTAAACCTCAACACCTTGTATCGTAACTAATATTTCTTGAGATAAGCACACTGCACCCATACCTTCCTTAAAAACGTAGCTTCCAGTTTTTGGTGGTTCCGGCTTCCTTCCCGATTCCGCCCGCTAAACGCATGTTTTTGTTGCCTGGTGGCATTTGCAAAATGCATAACCTATGCATTTAAAAGATTATGTATGCTCTTCTGACTTTTCGTGTGATGAGGCTCGTGGAAAAAATGAGTAATTTATGAACTTGAGAACAATTTTGTGTTGTTACGGTACTTTACTATGGAATAATCAATCAATTGAGGATTTTGTATAAATATCGTTTGAATATTTCTCCGACCCTTTGAGTACTTTTCTTCATAATTGCATAATATTGTCCGCTGCCCCTTTTTCTGTTAGACGGCGTCTCGATCTACTTGCTGTCGTTCAACACCACCTTATTTTCTAACTATTTTTTTTTTAGCTCATTTGAATCGGCTTATGGTGATGGCACGTTTTTGCATAAACCTAGCTGTCCTCGTTGAACATAGGAAAAAAAAATATATAAACAAGGCTCTTTCACTCTCCTTGCAATCAGATTTGGGTTTGTTCCCTTTATTTTCATATTTCTTGCCATATTCCTTTCTCAATTATTATTTTCTACTTATAACCTCACGCAAAATAGCACAGTCAAATCAGTCAAG |


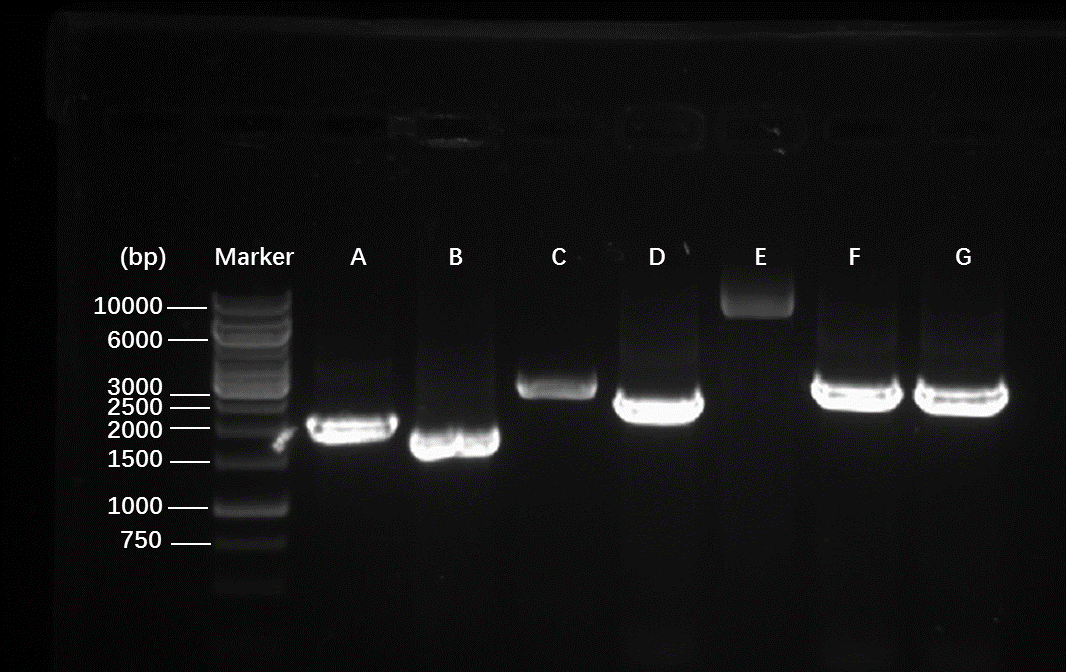


**Figure S1.** DNA electrophoresis confirmed that all of the cassettes in both xylose pathway and 1-hexadecanol pathway existed in the evolved strains (second generation). (A) XR cassette (2160 bp) in pXF3X03; (B) XDH cassette (1730 bp) in pXF3X03; (C) XK cassette (2800 bp) in in pXF3X03; (D) TaFAR cassette (2503 bp) in pTaFAR_ACC1; (E) ACC1 cassette (7615 bp) in pTaFAR_ACC1; (F) ACL cassette1 (2840 bp) in pYlACL; (G) ACL cassette2 (2850 bp) in pYlACL.





**Figure S2.** The strengths of PDC1, TEF1, and ENO2 promoters in front of EGFP. The strength of wild-type PDC1, TEF1, and ENO2 promoter was normalized into 100. Black, red, and blue bars represent the promoters with low, medium, and high strength, respectively.


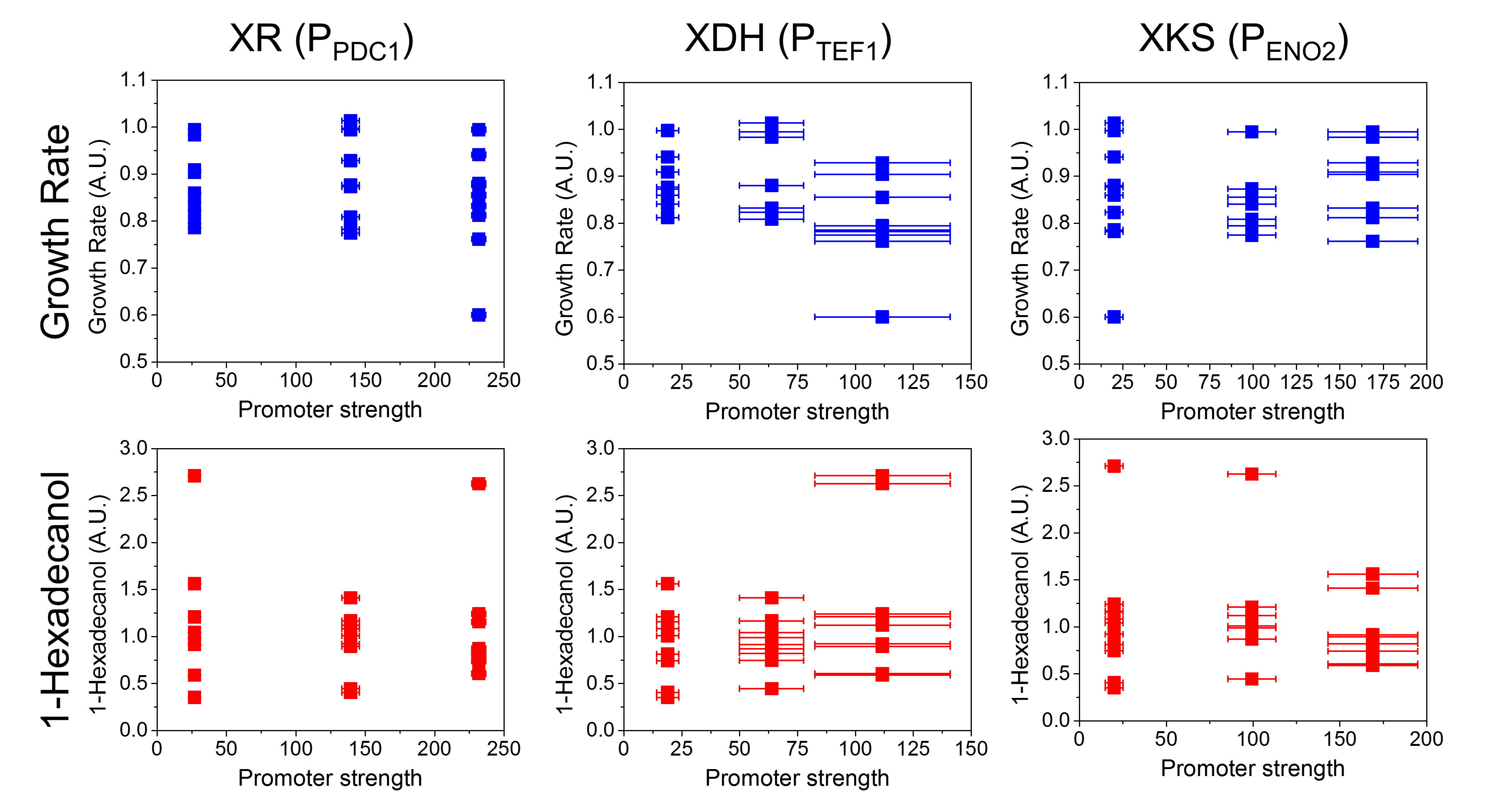


**Figure S3.** Correlations between the promoter strengths in front of XR, XDH and XKS in xylose utilization pathways and the growth rates (blue dots) as well as 1-hexadecanol concentrations (red dots). The unit of promoter strength was the same as that was used in Figure S2, i.e., normalized fluorescence (A.U.) of EGFP expressed by PDC1, TEF1 and ENO2 promoters, respectively. No correlation was observed between promoter strength and 1-hexadecanol concentration. Similarly, no correlation was observed between promoter strength and growth.

**Figure S4.** Correlation between the growth rates and the 1-hexadecanol titers for the combinatorial promoter engineering.

C

B

A


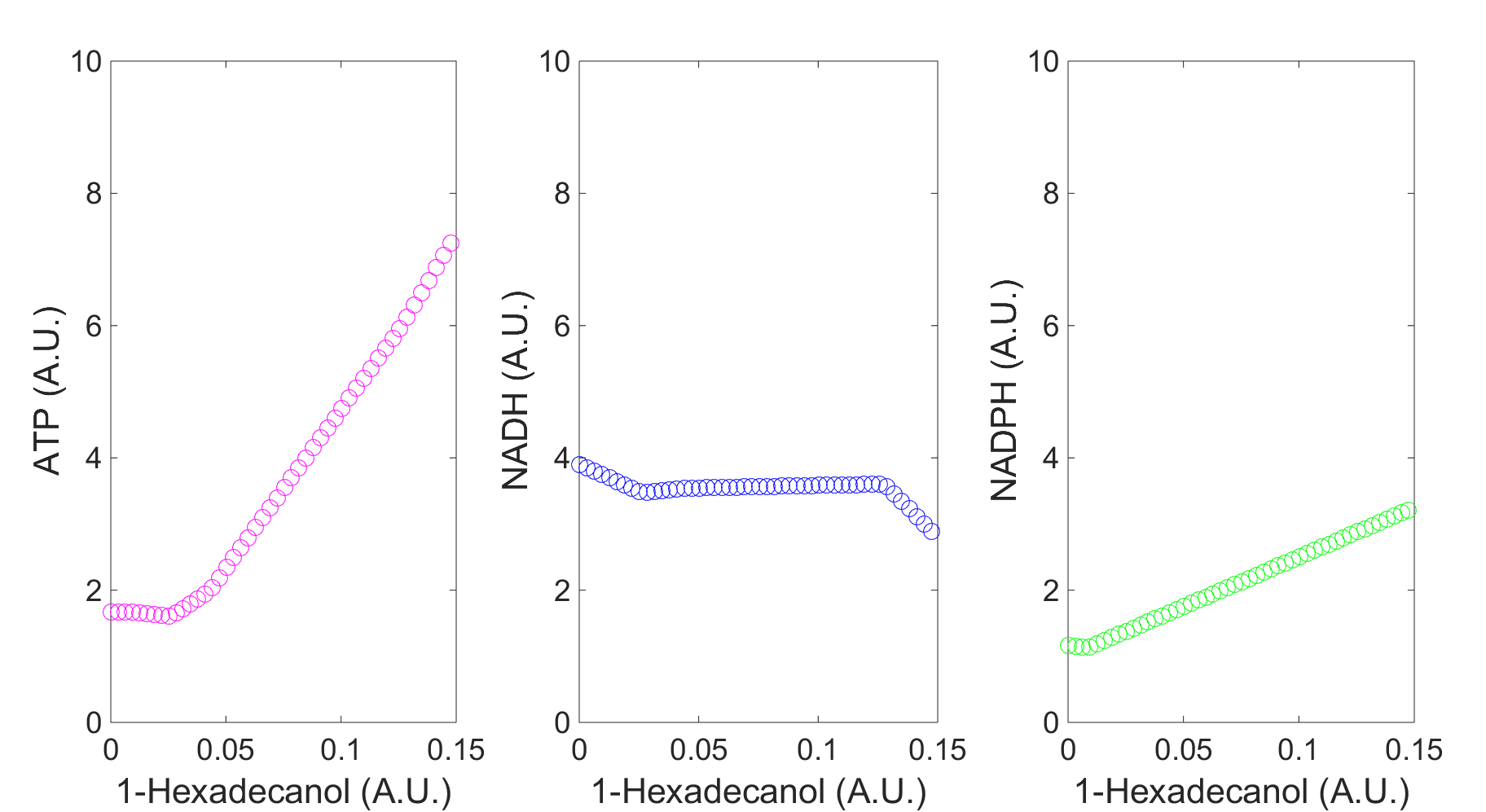


D


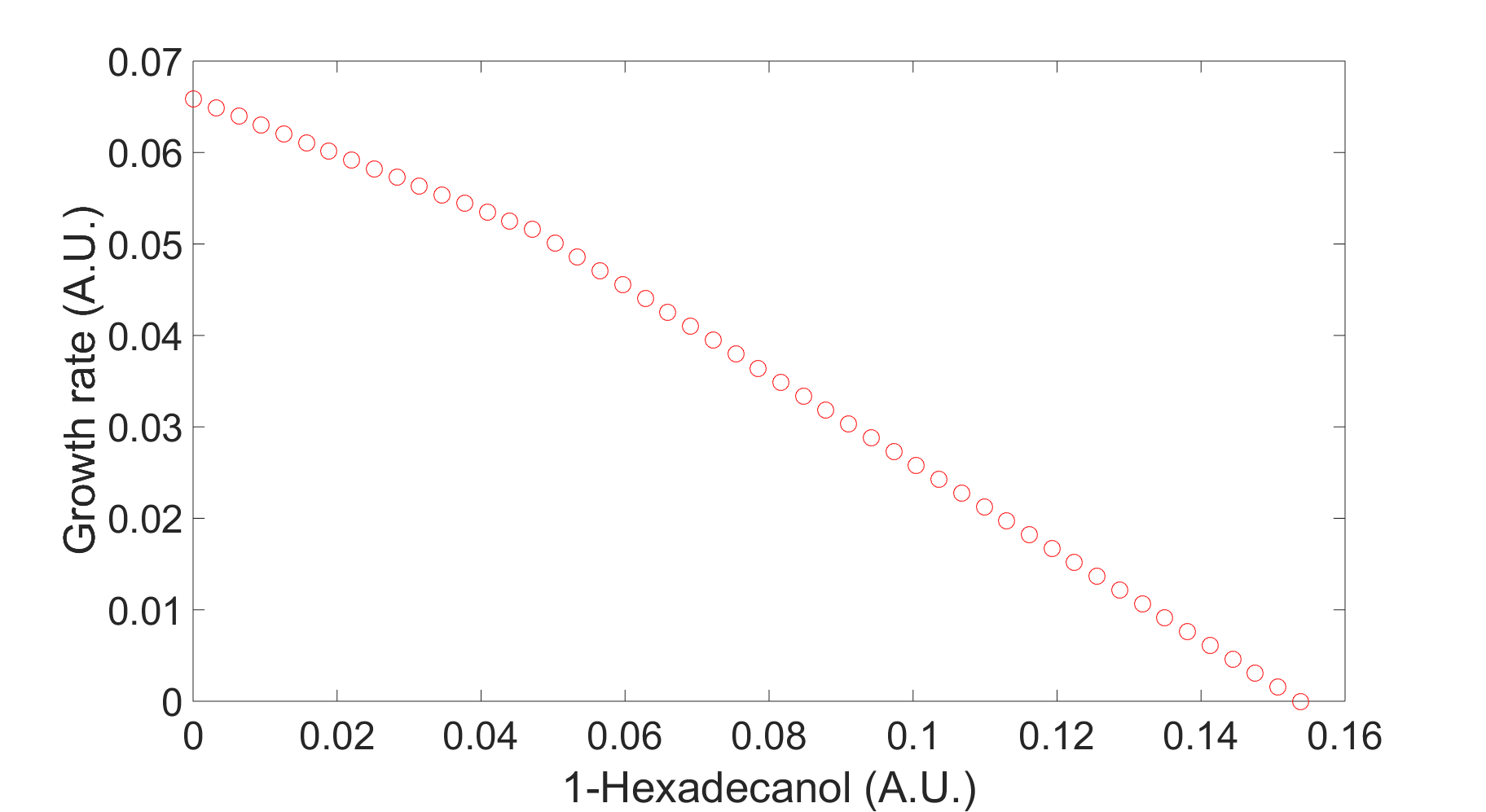


**Figure S5.** Flux balance analysis revealed correlation between the 1-hexadecanol production and ATP (A), NADH (B), NADPH (C), and growth rate (D).
